# Global Systems for Mobile Position Tracking Using Kalman and Lainiotis Filters

**DOI:** 10.1155/2014/130512

**Published:** 2014-02-23

**Authors:** Nicholas Assimakis, Maria Adam

**Affiliations:** ^1^Department of Electronic Engineering, Technological Educational Institute of Central Greece, 3rd km Old National Road Lamia-Athens, 35100 Lamia, Greece; ^2^Department of Computer Science and Biomedical Informatics, University of Thessaly, 2-4 Papasiopoulou Street, 35100 Lamia, Greece

## Abstract

We present two time invariant models for Global Systems for Mobile (GSM) position tracking, which describe the movement in *x*-axis and *y*-axis simultaneously or separately. We present the time invariant filters as well as the steady state filters: the classical Kalman filter and Lainiotis Filter and the Join Kalman Lainiotis Filter, which consists of the parallel usage of the two classical filters. Various implementations are proposed and compared with respect to their behavior and to their computational burden: all time invariant and steady state filters have the same behavior using both proposed models but have different computational burden. Finally, we propose a Finite Impulse Response (FIR) implementation of the Steady State Kalman, and Lainiotis filters, which does not require previous estimations but requires a well-defined set of previous measurements.

## 1. Introduction

The Global Positioning System (GPS) is the most popular positioning technique in navigation providing reliable mobile location estimates in many applications [1–4]. Thus wireless location systems offering reliable mobile location estimates have been studied by researchers and engineers over the past few years. Various techniques require one base station or at least two base stations or more than three base stations in order to determine the location of the user. The accuracy of the positioning results is affected by many interference sources as the signals propagate in the atmosphere. So, techniques were developed using filters to estimate the location of the user through the location information exchanged between the handset and the base station. Kalman filter has been used in the localization process [4–6], due to the following advantages mentioned in [5]: (a) Kalman filter [7–9] processes noisy measurements and so it can smooth out the effects of noise in the estimated state variables by integrating more information from reliable data more than unreliable data and (b) Kalman filter allows the combination of measurements from different sources (locomotion data) and different times. Kalman filter was implemented for Global Systems for Mobile (GSM) position tracking in [5]: Kalman filter was used for tracking in two dimensions and it was stated that Kalman filter is very powerful due to its reliable performance, because it yielded enhanced position tracking results.

In this paper we extend the ideas in [5] in two fields: (a) by using two models for GSM position tracking, which describe the movement in *x*-axis and *y*-axis simultaneously or separately and (b) by using the Kalman filter and the Lainiotis filter [8, 10]. The paper is organized as follows. In Section 2, we present two time invariant models for Global Systems for Mobile (GSM) position tracking, which describe the movement in *x*-axis and *y*-axis. In Section 3, we present the time invariant filters: Kalman filter, Lainiotis Filter and Join Kalman Lainiotis Filter. In Section 4, we present the corresponding steady state filters. In Section 5, various implementations are proposed. In Section 6, we compare the filters with respect to their behavior and to their computational burden. In Section 7, we propose a Finite Impulse Response (FIR) implementation of the Steady State Kalman and Lainiotis Filters. Finally, Section 8 summarizes the conclusions.

## 2. Time Invariant Models

Linear estimation is associated with time invariant systems described by the following state space equations:

(1) x(k+1)=Fx(k)+Gw(k),z(k)=Hx(k)+v(k)

for *k* ≥ 0, where *x*(*k*) is the *n*-dimensional state vector at time *k*, *z*(*k*) is the *m*-dimensional measurement vector at time *k*, *F* is the *n* × *n* system transition matrix, *H* is the *m* × *n* output matrix, *w*(*k*) is the plant noise at time *k*, *v*(*k*) is the measurement noise at time *k*. Also, {*w*(*k*)} and {*v*(*k*)} are Gaussian zero-mean white random processes with covariance matrices *Q* and *R*, respectively. The initial state *x*(0) is a Gaussian random variable with mean *x*_0_ and covariance *P*_0_ and is assumed to be independent of *w*(*k*) and *v*(*k*).

In this paper we consider two models.

*Model A.* The first model (model A) describes the movement in *x*-axis and *y*-axis simultaneously and follows the ideas in [5].

The state vector is of dimension *n* = 4 and contains the position and the velocity in *x*-axis and *y*-axis: *x*(*k*) = [*s*_*x*_(*k*) *υ*_*x*_(*k*) *s*_*y*_(*k*) *υ*_*y*_(*k*)]^*T*^. The measurement vector is of dimension *m* = 2 and contains the measured position *x*-axis and *y*-axis: *z*(*k*) = [*z*_*x*_(*k*) *z*_*y*_(*k*)]^*T*^.

Then we take:

(2) $$\begin{array}{c} F=\left[\begin{array}{cccc} 1 & \Delta t & 0 & 0\\ 0 & 1 & 0 & 0\\ 0 & 0 & 1 & \Delta t\\ 0 & 0 & 0 & 1 \end{array}\right],\;\;G=\left[\begin{array}{cc} \frac{1}{2}\Delta t & 0\\ 1 & 0\\ 0 & \frac{1}{2}\Delta t\\ 0 & 1 \end{array}\right],\\ H=\left[\begin{array}{cccc} 1 & \Delta t & 0 & 0\\ 0 & 0 & 1 & \Delta t \end{array}\right]. \end{array}$$

The plant noise *w*(*k*) = [*w*_*x*_(*k*) *w*_*y*_(*k*)]^*T*^ is Gaussian zero-mean with covariance matrix $Q=\left[\begin{array}{cc} \sigma_{xq}^{2} & 0\\ 0 & \sigma_{yq}^{2} \end{array}\right]$.

The measurement noise *v*(*k*) = [*v*_*x*_(*k*) *v*_*y*_(*k*)]^*T*^ is Gaussian zero-mean with covariance matrix $=\left[\begin{array}{cc} \sigma_{xr}^{2} & 0\\ 0 & \sigma_{yr}^{2} \end{array}\right]$.

*Model B.* The second model (model B) describes the movement in *x*-axis and *y*-axis separately. In each axis, the state vector is of dimension *n* = 2 and contains the position and the velocity: *x*(*k*) = [*s*(*k*) *υ*(*k*)]^*T*^. The measurement vector vector is of dimension *m* = 1 and contains the measured position *z*(*k*).

Then we take:

(3) $$\begin{array}{c} F=\left[\begin{array}{cc} 1 & \Delta t\\ 0 & 1 \end{array}\right],\;\;G=\left[\begin{array}{c} \frac{1}{2}\Delta t\\ 1 \end{array}\right],\;\;H=\left[\begin{array}{cc} 1 & \Delta t \end{array}\right]. \end{array}$$

The plant noise *w*(*k*) is Gaussian zero-mean with covariance matrix *Q* = *σ*_*q*_^2^.

The measurement noise *v*(*k*) is Gaussian zero-mean with covariance matrix = *σ*_*r*_^2^.

It is obvious that we are able to describe the movement in both axes using two separate state vectors: *x*_*x*_(*k*) = [*s*_*x*_(*k*) *υ*_*x*_(*k*)]^*T*^ for the *x*-axis and *x*_*y*_(*k*) = [*s*_*y*_(*k*) *υ*_*y*_(*k*)]^*T*^ for the *y*-axis. If we merge these two state vectors, we take the state vector *x*(*k*) = [*s*_*x*_(*k*) *υ*_*x*_(*k*) *s*_*y*_(*k*) *υ*_*y*_(*k*)]^*T*^ of model A.

## 3. Time Invariant Kalman and Lainiotis Filters

In this section, we present the classical time invariant Kalman filter [7–9] and Lainiotis Filter [8, 10], which are the most well-known algorithms that solve the filtering problem. Both algorithms compute the estimation *x*(*k*/*k*) and the corresponding estimation error covariance *P*(*k*/*k*). We also propose the Join Kalman-Lainiotis Filter which consists of the parallel (with the same measurements) usage of* two* filters (one Kalman filter and one Lainiotis Filter) and combination of the results (weight 50% for each filter).

*Kalman Filter (KF).* The following equations constitute the KF:

(4) x(k+1/k)=Fx(k/k),P(k+1/k)=(GQGT)+FP(k/k)FT,K(k+1)=P(k+1/k)HT[HP(k+1/k)HT+R]−1,x(k+1/k+1)=[I−K(k+1)H]x(k+1/k) +K(k+1)z(k+1),P(k+1/k+1)=[I−K(k+1)H]P(k+1/k),

for *k* ≥ 0, with initial conditions *x*(0/0) = *x*_0_ and *P*(0/0) = *P*_0_.

The Kalman filter computes the estimation *x*(*k*/*k*) and the estimation error covariance *P*(*k*/*k*) through the prediction *x*(*k* + 1/*k*) and the corresponding prediction error covariance *P*(*k* + 1/*k*) using the Kalman filter gain *K*(*k*).

*Lainiotis Filter (LF).* The following equations constitute the LF:

(5) x(k+1/k+1)=Knz(k+1)+Fn[I+P(k/k)On]−1 ×[P(k/k)Kmz(k+1)+x(k/k)],

(6) $$\begin{array}{c} P\left(k+1/k+1\right)=P_{n}+F_{n}{\left[I+P\left(k/k\right)O_{n}\right]}^{-1}P\left(k/k\right)F_{n}^{T} \end{array}$$

for *k* ≥ 0, with initial conditions *x*(0/0) = *x*_0_ and *P*(0/0) = *P*_0_, where

(7) $$\begin{array}{c} A={\left[H\left(GQG^{T}\right)H^{T}+R\right]}^{-1},\\ K_{n}=\left(GQG^{T}\right)H^{T}A,\\ K_{m}=F^{T}H^{T}A,\\ P_{n}=\left(GQG^{T}\right)-K_{n}H\left(GQG^{T}\right),\\ F_{n}=F-K_{n}HF,\\ O_{n}=F^{T}H^{T}AHF. \end{array}$$

*Join Kalman-Lainiotis Filter (JKLF)*. The filter consists of the parallel usage of* two* filters (one Kalman filter and one Lainiotis Filter) with the same measurements and combination of the results (weight 50% for each filter):

(8) $$\begin{array}{c} x\left(k/k\right)=\frac{1}{2}x_{\text{KF}}\left(k/k\right)+\frac{1}{2}x_{\text{LF}}\left(k/k\right),\\ P\left(k/k\right)=\frac{1}{2}P_{\text{KF}}\left(k/k\right)+\frac{1}{2}P_{\text{LF}}\left(k/k\right). \end{array}$$

## 4. Steady State Kalman and Lainiotis Filters

For time invariant systems, it is well known [7] that there exists a steady state value *P*_*p*_ of the prediction error covariance matrix, if the signal process model is asymptotically stable, or if the signal process model is not necessarily asymptotically stable, but the pair [*F*, *H*] is completely detectable and the pair [*F*, *GG*_1_] is completely stabilizable for any *G*_1_ with *G*_1_ *G*_1_^*T*^ = *Q*. Then there also exist a steady state value *P*_*e*_ of the estimation error covariance matrix and a steady state value *K* of the Kalman filter gain.

In this section we present the Steady State Kalman filter and Lainiotis Filter. Both algorithms compute the estimation *x*(*k*/*k*) using the previous estimation and the current measurement. We also propose the Join Steady State Kalman-Lainiotis Filter, which consists of the parallel usage of *two* filters (one Steady State Kalman filter and one Steady State Lainiotis Filter) with the same measurements and combination of the results (weight 50% for each filter).

*Steady State Kalman Filter (SSKF). *The following equation constitutes the SSKF:

(9) $$\begin{array}{c} x\left(k+1/k+1\right)=A_{\text{KF}}x\left(k/k\right)+B_{\text{KF}}z\left(k+1\right) \end{array}$$

for *k* ≥ 0, with initial condition *x*(0/0) = *x*_0_, where

(10) $$\begin{array}{c} A_{\text{KF}}=\left[I-KH\right]F,\\ B_{\text{KF}}=K. \end{array}$$

The steady state Kalman filter gain *K* is computed by *K* = *P*_*p*_ *H* ^*T*^[*HP*_*p*_ *H* ^*T*^ + *R*]^−1^, where *P*_*p*_ is the steady state prediction error covariance computed by solving the* Riccati equation emanating from Kalman filter (REKF):*

(11) $$\begin{array}{c} P_{p}=\left(GQG^{T}\right)+\text{F}{\text{P}}_{p}F^{T}-\text{F}{\text{P}}_{p}H^{T}{\left[\text{H}{\text{P}}_{p}H^{T}+R\right]}^{-1}\text{H}{\text{P}}_{p}F^{T}. \end{array}$$

In view of the importance of the Riccati equation emanating from Kalman filter, there exists considerable literature on its algebraic solutions [7, 11] or iterative solutions [7, 12–15] concerning per step or doubling algorithms.

*Steady State Lainiotis Filter (SSLF).* The following equation constitutes the SSLF:

(12) $$\begin{array}{c} x\left(k+1/k+1\right)\;=A_{\text{LF}}x\left(k/k\right)+B_{\text{LF}}z\left(k+1\right), \end{array}$$

for *k* ≥ 0, with initial condition *x*(0/0) = *x*_0_, where

(13) $$\begin{array}{c} A_{\text{LF}}=F_{n}{\left[I+P_{e}O_{n}\right]}^{-1},\\ B_{\text{LF}}=K_{n}+F_{n}{\left[I+P_{e}O_{n}\right]}^{-1}P_{e}K_{m}, \end{array}$$

and *P*_*e*_ is the steady state estimation error covariance computed by solving the* Riccati equation emanating from Lainiotis filter (RELF):*

(14) $$\begin{array}{c} P_{e}=P_{n}+F_{n}{\left[I+P_{e}O_{n}\right]}^{-1}P_{e}F_{n}^{T}. \end{array}$$

In view of the importance of the Riccati equation emanating from Lainiotis Filter, there exists considerable literature on its algebraic or iterative solutions [12, 14–16] concerning per step or doubling algorithms.

Note that in [8] it is shown that SSKF is equivalent to SSLF, since

(15) $$\begin{array}{c} A_{\text{KF}}=A_{\text{LF}},\\ B_{\text{KF}}=B_{\text{LF}}. \end{array}$$

*Join Steady State Kalman-Lainiotis Filter (JSSKLF).* The filter consists of the parallel usage of* two* steady state filters (one Steady State Kalman filter and one Steady State Lainiotis Filter) with the same measurements and combination of the results (weight 50% for each filter):

(16) $$\begin{array}{c} x\left(k/k\right)=\frac{1}{2}x_{\text{KF}}\left(k/k\right)+\frac{1}{2}x_{\text{LF}}\left(k/k\right), \end{array}$$

for *k* ≥ 0.

## 5. Implementations

In this section, we propose various implementations.

The use of model A which describes the movement in *x*-axis and *y*-axis simultaneously requires the use one filter; we are able to use KF/LF/SSKF/SSLF/JKLF in order to compute the estimation and the corresponding estimation error covariance.

The use of model B, which describes the movement in *x*-axis and *y*-axis separately, requires the use of two filters KF/LF/SSKF/SSLF/JSSKLF in order to compute the estimation and the corresponding estimation error covariance for each movement. It is obvious that, if we merge the estimation *x*_*x*_(*k*/*k*) = [*s*_*x*_(*k*/*k*) *υ*_*x*_(*k*/*k*)]^*T*^ for the movement in *x*-axis and the estimation *x*_*y*_(*k*/*k*) = [*s*_*y*_(*k*/*k*) *υ*_*y*_(*k*/*k*)]^*T*^ for the movement in *y*-axis, we take the state vector of model A:

(17) x(k/k)=[sx(k/k)υx(k/k)sy(k/k)υy(k/k)]T=[xx(k/k)xy(k/k)]T.

Also, the estimation error covariances *P*_*x*_(*k*/*k*) and *P*_*y*_(*k*/*k*) for each movement can be merged to the estimation error covariance of model A:

(18) $$\begin{array}{c} P\left(k/k\right)=\left[\begin{array}{cc} P_{x}\left(k/k\right) & 0\\ 0 & P_{y}\left(k/k\right) \end{array}\right]. \end{array}$$

Thus, we propose various implementations for Global Systems for Mobile (GSM) position tracking, as it is shown in Table 1.

## 6. Comparison of the Filters

In this section we compare the filters with respect to their behavior and to their computational burden.

**Example 1**

We implemented the filters with the following parameters:

- discretization factor: Δ*t* = 1,
- movement reliability: *σ*_*xq*_^2^ = *σ*_*yq*_^2^ = 0.01,
- measurements reliability: *σ*_*xr*_^2^ = *σ*_*yr*_^2^ = 0.1,
- initial conditions: *x*_0_ = 0 and *P*_0_ = 0.

Concerning the behavior of the filters, we found that

- the time invariant filters KF, LF and JKLF are equivalent, since they compute the same outputs (estimation and estimation error covariance), using model A or model B,
- the steady state filters SSKF, SSLF and JSSKLF are equivalent, since they compute the same outputs (estimation and estimation error covariance), using model A or model B,
- the steady state filters and the time invariant filters compute outputs very close to each other,
- model A is equivalent to model B, since they produce the same outputs.

**Figure 1:**
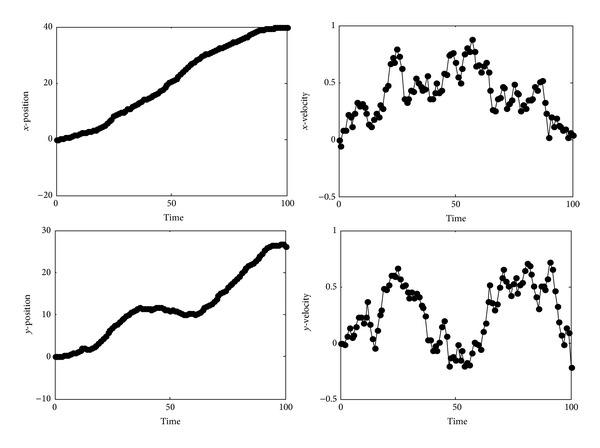
Position and velocity estimation solid line: KF/LF/JKLF, dashed line: SSKF/SSLF/JSSKLF.

These results are depicted in Figure 1.

Concerning the computational burden of the filters, we compared the filters with respect to their per-iteration calculation burdens, computed using the ideas in [8], as shown in Table 2.

Table 3 summarizes the per-iteration calculation burden of all implementations, using model A and model B.

We observe that

- KF is faster than LF,
- JKLF is slower than KF and LF (since the join filter requires the implementation of both the Kalman and Lainiotis filters),
- SSKF is as fast as SSLF,
- SSKF and SSLF are faster than KF and LF,
- JSSKLF is slower than SSKF and SSLF,
- the filters using model B are faster than the same filters using model A.

Table 4 summarizes speedup between the various implementations.

We observe that

(i) KF is faster than LF,
   speedup (LF model A to KF model A) = 1.316,
   speedup (LF model B to KF model B) = 1.290.
(ii) Model B is faster than model A,
   speedup (KF model A to SSKF model B) = 25.450,
   speedup (LF model A to SSLF model B) = 33.500.

## 7. FIR Steady State Kalman and Lainiotis Filters

In this section we propose an FIR implementation of the Steady State Kalman filter and the Steady State Lainiotis Filter.

Recall that SSKF and SSLF have equal parameters:

(19) $$\begin{array}{c} A=A_{\text{KF}}=A_{\text{LF}},\;\;B=B_{\text{KF}}=B_{\text{LF}}. \end{array}$$

Then we are able to write

(20) $$\begin{array}{c} x\left(k+1/k+1\right)=Ax\left(k/k\right)+Bz\left(k+1\right), \end{array}$$

for *k* ≥ 0 with initial condition *x*(0/0) = *x*_0_.

Then we take:

(21) x(1/1)=Ax0+Bz(1)x(2/2)=A2x0+ABz(1)+Bz(2)⋮x(ν/ν)=Aνx0+Aν−1Bz(1)+⋯+ABz(ν−1)+Bz(ν).

Using the ideas in [17], the resulting FIR SSKF/SSLF is formulated as

(22) $$\begin{array}{c} x\left(k/k\right)=\overset{M-1}{\underset{j=0}{\sum }}A^{j}Bz\left(k-j\right),\;\text{for}\;\;k\geq 1, \end{array}$$

where *A* = *A*_KF_ = *A*_LF_ and *B* = *B*_KF_ = *B*_LF_ and *M* is the FIR filter order defined by ||*A* ^*M*^|| < *ε* and ||*A* ^*M*−1^|| ≥ *ε*, with *ε* a small real value.

**Remark 1 s**

(1) The FIR steady state filter coefficients can be calculated off-line by solving the corresponding Riccati equation.

(2) The FIR steady state filter does not require previous estimations but it requires a well-defined set of *M* previous measurements. This means that we have to wait for *M* time moments in order to produce the results. Alternatively, we are able to use only the available measurements until time *M* is reached or to use SSKF until time *M*.

We implemented the FIR filter for the parameters of the example in Section 6. We used *ε* = 10^−3^ and we found *M* = 17.

**Figure 2:**
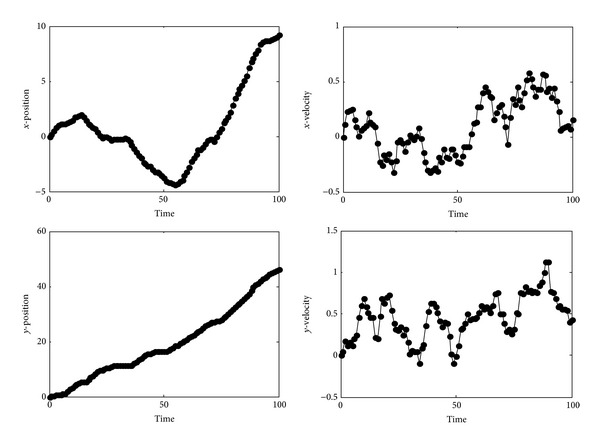
Position and velocity estimation solid line: SSKF/SSLF, dashed line: FIR SSKF/SSLF.

The steady state filters and FIR steady state filters compute outputs very close to each other, as depicted in Figure 2.

Concerning the computational burden, the FIR steady state filter possesses a constant burden while the classical steady state filter (SSKF/SSLF) possesses a constant per-iteration computational burden, as it is shown in Table 5.

Table 6 summarizes the calculation burden of the classical and FIR steady state filters implementations, using model A and model B.

Thus, FIR SSKF/SSLF can be faster than SSKF/SSLF, if we take results using FIR SSKF/SSLF every (or more than) *μ* time lags, where *μ* is the nearest integer greater than or equal to the ratio (40 + 4*M*)/44 = (10 + *M*)/11 for model A and (16 + 4*M*)/20 = (4 + *M*)/5 for model B.

For our example, we take the results, which are appeared in Table 7.

## 8. Conclusions

In this paper we presented two time invariant models for Global Systems for Mobile (GSM) position tracking, which describe the movement in *x*-axis and *y*-axis simultaneously or separately. We presented the time invariant filters as well as the steady state filters: the classical Kalman filter and Lainiotis Filter and the Join Kalman Lainiotis Filter, which consists of the parallel usage of the two classical filters. Various implementations are proposed and compared with respect to their behavior and to their computational burden. We found that all time invariant and steady state filters have the same behavior using both of the proposed models. We found that (a) Kalman filter is faster than Lainiotis Filter, (b) Join Kalman Lainiotis Filter is slower than both Kalman filter and Lainiotis Filter, (c) steady state filters are faster than time invariant filters and (d) the filters using the model, which handles the movement in *x*-axis and *y*-axis separately, are faster than the same filters using the model, which handles the movement in *x*-axis and *y*-axis simultaneously. Finally, we proposed an FIR implementation of the Steady State Kalman and Lainiotis Filters, which does not require previous estimations but it requires a well-defined set of previous measurements.

## Figures and Tables

**Table 1:** GSM position tracking implementations.

Implementation	Model	System	Filter
1	Model A	Time invariant	KF
2			LF
3			JKLF
4		Steady state	SSKF
5			SSLF
6			JSSKLF

7	Model B	Time invariant	KF
8			LF
9			JKLF
10		Steady state	SSKF
11			SSLF
12			JSSKLF

**Table 2:** Per-iteration calculation burden of filters.

KF	4*n* ^3^ + 3.5*n* ^2^ − 1.5*n* + 4*n* ^2^ *m* + *nm* + 3*nm* ^2^ + (16*m* ^3^ − 3*m* ^2^ − *m*)/6
LF	4*nm* + (58*n* ^3^ + 9*n* ^2^ − 7*n*)/6
JKLF	*n* ^2^ + 3*n* (join procedure)
SSKF	2*n* ^2^ + 2*nm* − *n*
SSLF	2*n* ^2^ + 2*nm* − *n*
JSSKLF	2*n* (join procedure)

**Table 3:** Per-iteration calculation burden of implementations.

Implementation	Model	System	Filter	Calculation burden
1	Model A	Time invariant	KF	509
2			LF	670
3			JKLF	1207
4		Steady state	SSKF	44
5			SSLF	44
6			JSSKLF	96

7	Model B	Time invariant	KF	138
8			LF	178
9			JKLF	326
10		Steady state	SSKF	20
11			SSLF	20
12			JSSKLF	44

**Table 4:** Speedup.

			1	2	3	4	5	6	7	8	9	10	11	12
			Model A						Model B					
			KF	LF	JKLF	SSKF	SSLF	JSSKLF	KF	LF		SSKF	SSLF	JSSKLF
1	Model A	KF		0,760	0,427	11,568	11,568	4,314	3,688	2,860	0,795	25,450	25,450	5,194
2		LF	1,316		0,563	15,227	15,227	5,678	4,855	3,764	1,047	33,500	33,500	6,837
3		JKLF	2,340	1,778		27,068	27,068	10,093	8,630	6,691	1,861	59,550	59,550	12,153
4		SSKF	0,086	0,066	0,037		1,000	0,373	0,319	0,247	0,069	2,200	2,200	0,449
5		SSLF	0,086	0,066	0,037	1,000		0,373	0,319	0,247	0,069	2,200	2,200	0,449
6		JSSKLF	0,232	0,176	0,099	2,682	2,682		0,855	0,663	0,184	5,900	5,900	1,204

7	Model B	KF	0,271	0,206	0,116	3,136	3,136	1,169		0,775	0,216	6,900	6,900	1,408
8		LF	0,350	0,266	0,149	4,045	4,045	1,508	1,290		0,278	8,900	8,900	1,816
9		JKLF	1,257	0,955	0,537	14,545	14,545	5,424	4,638	3,596		32,000	32,000	6,531
10		SSKF	0,039	0,030	0,017	0,455	0,455	0,169	0,145	0,112	0,031		1,000	0,204
11		SSLF	0,039	0,030	0,017	0,455	0,455	0,169	0,145	0,112	0,031	1,000		0,204
12		JSSKLF	0,193	0,146	0,082	2,227	2,227	0,831	0,710	0,551	0,153	4,900	4,900	

**Table 5:** Calculation burden of classical and FIR Steady State filters.

SSKF/SSLF	2*n* ^2^ + 2*nm* − *n*	Per-iteration
FIR SSKF/SSLF	2*n* ^2^ + 2*nm* − *n* + (*M* − 1)*n*	When required

**Table 6:** Calculation burden of classical and FIR steady state filters implementations.

	Model A	Model B
SSKF/SSLF	44	20
FIR SSKF/SSLF	40 + 4*M*	16 + 4*M*

**Table 7:** FIR results for Example 1.

M = 17	Model A	Model B
SSKF/SSLF	44	20
FIR SSKF/SSLF	108	84
*μ*	3	5
